# The use of venoarterial extracorporeal membrane oxygenation in cardiogenic shock: a narrative review

**DOI:** 10.1093/ehjopen/oeae051

**Published:** 2024-06-16

**Authors:** Tara Gédéon, Tetiana Zolotarova, Mark J Eisenberg

**Affiliations:** Division of Cardiology, Jewish General Hospital, McGill University, 3755 Côte Ste-Catherine Road, Suite H-421.1, Montreal, QC H3T 1E2, Canada; Centre for Clinical Epidemiology, Lady Davis Institute for Medical Research, Jewish General Hospital, 3755 Côte Ste-Catherine Road, Montreal, QC H3T 1E2, Canada; Division of Cardiology, Jewish General Hospital, McGill University, 3755 Côte Ste-Catherine Road, Suite H-421.1, Montreal, QC H3T 1E2, Canada; Centre for Clinical Epidemiology, Lady Davis Institute for Medical Research, Jewish General Hospital, 3755 Côte Ste-Catherine Road, Montreal, QC H3T 1E2, Canada; Department of Medicine, McGill University, 1001 Decarie Boulevard, Suite D05-2212, Montreal, QC H4A 3J1, Canada; Department of Epidemiology, Biostatistics and Occupational Health, McGill University, 2001 McGill College Ave, Montreal, QC H3A 1Y7, Canada

**Keywords:** VA-ECMO, Cardiogenic shock, Acute myocardial infarction, Mechanical circulatory support

## Abstract

**Aims:**

Cardiogenic shock (CS) develops in up to 10% of patients with acute myocardial infarction (AMI) and carries a 50% risk of mortality. Despite the paucity of evidence regarding its benefits, venoarterial extracorporeal membrane oxygenation (VA-ECMO) is increasingly used in clinical practice in patients with AMI in CS (AMI-CS). This review aims to provide an in-depth description of the four available randomized controlled trials to date designed to evaluate the benefit of VA-ECMO in patients with AMI-CS.

**Methods and results:**

The literature search was conducted on PubMed, Google Scholar, and clinicaltrials.gov to identify the four relevant randomized control trials from years of inception to October 2023. Despite differences in patient selection, nuances in trial conduction, and variability in trial endpoints, all four trials (ECLS-SHOCK I, ECMO-CS, EUROSHOCK, and ECLS-SHOCK) failed to demonstrate a mortality benefit with the use of VA-ECMO in AMI-CS, with high rates of device-related complications. However, the outcome of these trials is nuanced by the limitations of each study that include small sample sizes, challenging patient selection, and high cross-over rates to the intervention group, and lack of use of left ventricular unloading strategies.

**Conclusion:**

The presented literature of VA-ECMO in CS does not support its routine use in clinical practice. We have yet to identify which subset of patients would benefit most from this intervention. This review emphasizes the need for designing adequately powered trials to properly assess the role of VA-ECMO in AMI-CS, in order to build evidence for best practices.

## Graphical Abstract

**Graphical Abstract:**
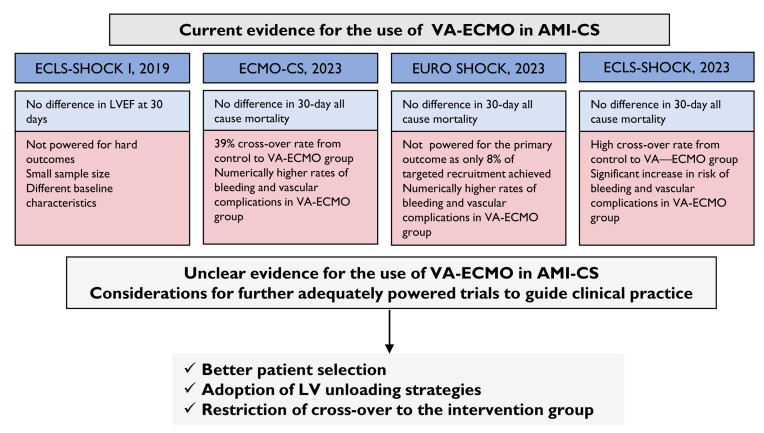
Current evidence for the use of VA-ECMO in AMI-CS and considerations for further trials to guide clinical practice. AMI-CS, acute myocardial infarction with cardiogenic shock; LV, left ventricle; LVEF, left ventricular ejection fraction; VA-ECMO, venoarterial extracorporeal membrane oxygenation.

## Introduction

Cardiogenic shock (CS) is a life-threatening condition that develops in up to 10% of hospitalized patients with acute myocardial infarction (AMI).^1^ It is defined clinically by hypotension and a low cardiac output state, leading to inadequate systemic tissue perfusion and end-organ damage.^1^ Significant improvement in outcomes has been attained due to early revascularization of the culprit lesion; reperfusion is to be achieved as soon as possible, preferably by percutaneous coronary intervention (PCI) or surgical intervention whenever possible.^1^ Nevertheless, rates of in-hospital mortality of patients with AMI in CS (AMI-CS) remain very high, reaching up to 50%.^2,3^ Venoarterial extracorporeal membrane oxygenation (VA-ECMO) is a temporary mechanical circulatory support (MCS) system that delivers the highest degree of cardiopulmonary support.^4^ Despite the paucity of evidence regarding its benefit in AMI-CS, its use has increased in clinical practice. Only four randomized control trials (RCTs) evaluating the benefits of VA-ECMO in patients with AMI-CS have been published. A recent patient-level meta-analysis of all four trials did not demonstrate a mortality benefit with the use of VA-ECMO in patients with AMI-CS, with an observed increased rate of bleeding and vascular complications.^5^ In this review, we provide an in-depth discussion of each trial, highlighting the outcomes and directing attention to the limitations and nuances of each study. We also emphasize the necessity of further trials to demonstrate the efficacy and safety of implantation of VA-ECMO in AMI-CS, to build evidence for best practices.

## Methods

The literature search was conducted to identify relevant RCTs reporting on the use of VA-ECMO in patients with AMI-CS. The databases searched to identify these four trials were PubMed, Google Scholar, and clinicaltrials.gov. The search period covered the years from inception to October 2023. Medical subject heading terms were used when relevant for the following key words: ‘VA-ECMO’, ‘extracorporeal life support’, ‘cardiogenic shock’, ‘acute myocardial infarction’, and ‘randomized controlled trials’. Relevant search results were identified through title screening or snowballing references of the included articles.

## Results

### ECLS-SHOCK I

The ECLS-SHOCK I trial by Brunner *et al*.^6^ was the first small randomized monocentral open-label controlled trial that evaluated the role of VA-ECMO in patients with AMI-CS. A total of 42 patients between 18 and 75 years of age were randomized to either VA-ECMO with standard therapy (*n* = 21) or standard therapy alone (*n* = 21). Patients who underwent cardiopulmonary resuscitation (CPR) for over 60 min or had evidence of infarct-related mechanical complications were excluded. Cardiogenic shock was defined as systolic blood pressure (SBP) <90 mmHg for over 30 min or the need of an infusion of catecholamine to maintain SBP >90 mmHg, with clinical signs of pulmonary congestion and evidence of impaired organ perfusion (*Table 1*). The study was powered to detect a difference in the primary endpoint of left ventricular ejection fraction (LVEF) assuming a 5% improvement in LVEF with VA-ECMO. Baseline characteristics in both groups were different with regard to a median age of 62 years [interquartile range (IQR) 50–68 years] in VA-ECMO group compared with 70 years (IQR 60–74 years) in the standard therapy group. The LVEF at 30 days was 50% in the VA-ECMO group (IQR 44.0–59.0%) and 50.8% in the standard therapy group (IQR 47.2–60.6%). The rates of safety secondary outcomes were not statistically different between the two groups and included rates of stroke (VA-ECMO 5% vs. control 5%; *P* = 1.0), peripheral ischaemic vascular complications (10 vs. 0%; *P* = 0.49), and bleeding complications (19 vs. 14%; *P* = 1.0). However, subgroup analysis of survivors only with respect to process-of-care outcomes showed significantly longer intensive care unit (ICU) stays (*P* = 0.003) and durations of mechanical ventilation (*P* < 0.01) for patients in the VA-ECMO group. Finally, 30-day all-cause mortality evaluated as a secondary outcome demonstrated a trend towards lower mortality in the VA-ECMO group without statistical significance (19 vs. 33%; *P* = 0.37), as the study was underpowered for that outcome (*Table 2*).

**Table 1:** Characteristics of randomized studies comparing venoarterial extracorporeal membrane oxygenation vs. standard treatment in patients in cardiogenic shock

Study	Sample size	Inclusion criteria	Major exclusion criteria	SCAI shock stage no. (%)	Intervention arm		Primary outcome	Conclusion
					VA-ECMO group	Control group		
ECLS-SHOCK I^6^	42	CS due to AMI with planned revascularization CS defined as SBP <90 mmHg for >30 min or inotropes required to maintain SBP >90 mmHg Signs of left heart insufficiency and pulmonary congestion Signs of impaired organ perfusion^a^	CPR duration >60 min Infarct-related mechanical complications	Not reported	VA-ECMO + early revascularization and optimal medical therapy	Early revascularization and optimal medical therapy	LVEF at 30 days	No difference in LVEF at 30 days between both groups
ECMO-CS^7^	117	CS due to different aetiologies CS defined as rapidly deteriorating^b^ or severe^c^ SCAI shock Stage D or E	Cardiac arrest survivors remaining comatose Suspicion for PE or cardiac tamponade as cause of CS PAD precluding femoral artery cannulation	Stage D—72 (62%) Stage D + E—45 (38%)	Immediate VA-ECMO + revascularization and optimal medical therapy	Early conservative strategy with downstream use of VA-ECMO in case of worsening haemodynamic status^d^	Composite of death from any cause, resuscitated circulatory arrest, and implementation of another MCS at 30 days	No difference in clinical outcomes with immediate VA-ECMO implantation compared with early conservative strategy with downstream use of VA-ECMO if worsening haemodynamic status
EURO-SHOCK^8^	35	CS due to AMI for at least 30 min after attempted revascularization CS defined as SBP <90 mmHg for >30 min or inotropes required to maintain SBP >90 mmHg with clinical signs of pulmonary congestion and impaired organ perfusion^a^	OHCA with pH <7 or with initiation of PCR >10 min of collapse Infarct-related mechanical complications PAD precluding VA-ECMO access	Not reported	VA-ECMO + early revascularization and optimal medical therapy + IABP permitted as LV unloading device	Early revascularization and optimal medical therapy + IABP permitted as a MCS	30-day all-cause mortality	No mortality benefit observed at 30 days with VA-ECMO implantation Limited sample size limits interpretation of trial results
ECLS-SHOCK^9^	417	CS due to AMI with planned revascularization CS defined as SBP <90 mmHg >30 min or inotropes required to maintain SBP >90 mmHg, arterial lactate >3 mmol/L and signs of impaired organ perfusion^a^	CPR >45 min before randomization Infarct-related mechanical complications PAD precluding VA-ECMO access	Stage C—215 (52%) Stage D—56 (13%) Stage E—146 (35%)	Early VA-ECMO therapy + early revascularization and optimal medical therapy	Early revascularization and optimal medical therapy with escalation to another MCS if deemed necessary and cross-over to VA-ECMO if haemodynamic deterioration^e^	30-day all-cause mortality	No mortality benefit observed at 30 days with empiric use of VA-ECMO

AMI, acute myocardial infarction; CPR, cardiopulmonary resuscitation; CS, cardiogenic shock; LVEF, left ventricular ejection fraction; MCS, mechanical circulatory support; OHCA, out-of-hospital cardiac arrest; PCI, percutaneous coronary intervention; PE, pulmonary embolism; SBP, systolic blood pressure; SCAI, Society for Cardiovascular Angiography and Interventions; VA-ECMO, venoarterial extracorporeal membrane oxygenation.

^a^Altered mental status; cold, clammy skin, and limbs; oliguria with urine output <30 mL/h or elevated arterial lactate level of >2 mmol/L.

^b^Progressive haemodynamic instability necessitating vasopressors to maintain mean arterial pressure (MAP) >50 mmHg and impaired LVEF.

^c^Haemodynamic: cardiac index <2.2 L/min/m^2^ + norepinephrine dose >0.1 μg/kg/min + dobutamine dose > 5 μg/kg/min or SBP <100 mmHg + norepinephrine dose > 0.2 μg/kg/min + dobutamine dose > 5 μg/kg/min + impaired LVEF. Metabolic: 2 consecutive lactate values ≥3 mmol/L or SvO_2_ values <50% with no improvement on steady doses of inotropes and/or vasopressors.

^d^Rise in lactate by 3 mmol/L compared with the lowest value in the past 24 h.

^e^Haemodynamic deterioration despite escalation of medical therapy and use of other MCS, defined as severe haemodynamic instability, an increase in arterial lactate of >3 mmol/L during a 6 h period, or an increase in vasopressor use by 50% from baseline to maintain a MAP >65 mmHg.

**Table 2:** Outcomes of randomized studies comparing venoarterial extracorporeal membrane oxygenation vs. standard treatment in patients with cardiogenic shock

Study	Total sample size, *n*	Sample size, *n*		30-day all-cause mortality *n* (%)		Stroke *n* (%)		Life-threatening bleeding complications *n* (%)	
		VA-ECMO	ST	VA-ECMO	ST	VA-ECMO	ST	VA-ECMO	ST
ECLS-SHOCK I^6^	42	21	21	4 (19)	7 (33)	1 (5)	1 (5)	4 (19)	3 (14)
ECMO-CS^7^	117	58	59	29 (50.0)	28 (47.5)	3 (5.2)	0 (0)	18 (31.0)	12 (20.3)
EURO-SHOCK^8^	35	17	18	7 (43.8)	11 (61.1)	0 (0)	2 (11)	5 (29.4)	1 (6)
ECLS-SHOCK^9^	417	209	208	100 (47.8)	102 (49.0)	8 (3.8)	6 (2.9)	49 (23.4)	20 (9.6)

Summary of primary outcome of 30-day all-cause mortality in each individual trials as well as secondary safety outcomes.

ST, standard therapy; VA-ECMO, venoarterial extracorporeal membrane oxygenation.

#### Commentary

The ECLS-SHOCK I trial failed to demonstrate a clinical benefit with VA-ECMO implantation with regard to LVEF. However, 95% of the patients underwent CPR prior to implantation. Thus, improvement in the LVEF in both groups may be attributed to known reversible transient contractile impairment and myocardial stunning improvement following the cardiac arrest. In a population facing a 50% mortality risk, it remains debatable whether a modest improvement in the LVEF truly represents a meaningful endpoint. While any recovery in LVEF is important, it is unlikely to translate into tangible clinical outcomes or a significant reduction in mortality.

In addition, study outcomes may have been influenced by the underlying variability in baseline characteristics, notably age and degree of affected coronary disease. While no increased risk of vascular or bleeding complications were noted in the VA-ECMO group, the study was underpowered to detect a difference in secondary outcomes. Moreover, longer ICU stay and duration of mechanical ventilation in survivors from the VA-ECMO group highlight that any potential benefit from VA-ECMO may be counterbalanced by risks related to device implantation. The numerical trend towards lower mortality rates in the VA-ECMO group motivated the development of other RCTs powered to assess survival as a primary outcome.

### Extracorporeal membrane oxygenation in cardiogenic shock (ECMO-CS)

The ECMO-CS trial by Ostadal *et al*.^7^ was a multicentre, open-label RCT conducted across four centres in Czech Republic that aimed to assess the efficacy of early VA-ECMO implantation in patients with rapidly deteriorating or severe CS based on the Society for Cardiovascular Angiography and Interventions (SCAI) shock classification. Rapidly deteriorating CS (SCAI shock Stages D and E) and severe CS (SCAI shock Stage D) were defined by haemodynamic and metabolic parameters described in *Table 1*. A total of 117 patients with CS due to different aetiologies were randomized to immediate VA-ECMO therapy (*n* = 58) or early conservative therapy (*n* = 59). In the early conservative group, downstream use of VA-ECMO was allowed in case of worsening haemodynamic status, defined as a rise in lactate by 3 mmol/L in comparison with the lowest value during the past 24 h. A sample size of 120 patients was required to detect a 50% reduction in the primary endpoint of composite of death from any cause, resuscitated circulatory arrest, and implementation of another MCS at 30 days. Analyses were performed according to the intention-to-treat principle.

Baseline characteristics were similar between both groups. The median age was 67 years in the immediate VA-ECMO group and 65 years in the conservative therapy group. A total of 73.5% of the patients were males. Acute myocardial infarction was the most common aetiology of CS in both arms, accounting for over 60% of cases, followed by chronic heart failure. Over 70% of patients were on mechanical ventilation. The primary endpoint of composite of death from any cause, resuscitated circulatory arrest, and implementation of another MCS at 30 days occurred in 63.8% of patients in the immediate VA-ECMO group compared with 71.2% of patients in the early conservative group [hazard ratio (HR) 0.72, 95% confidence interval (CI) 0.46–1.12]. There were no differences in individual components of that endpoint with regard to all-cause mortality (50 vs. 47.5%; HR 1.11) and resuscitated cardiac arrest (10.3 vs. 13.6%; HR 0.79), but fewer patients in the immediate VA-ECMO group required an additional MCS device (17.2 vs. 42.4%; HR 0.38). A total of 39% of patients (*n* = 23) in the initial conservative group required VA-ECMO support, of whom 52.2% (*n* = 12) died. The mean time from randomization to insertion of VA-ECMO in the early conservative arm was 1.9 days. A subgroup analysis of the 74 patients with AMI-CS showed comparable incidence of the primary endpoint (59.5 vs. 67.6%, risk difference −8.1, 95% CI −30 to 13.8) and all-cause death (48.6 vs. 43.2%, risk difference 5.4, 95% CI −17.3 to 28.1) in the immediate VA-ECMO group and initial conservative therapy group, respectively. Although numerically higher in the immediate VA-ECMO group, secondary safety endpoints of clinically significant bleeding, leg ischaemia, and technical complications were not statistically different between both groups. Serious adverse events occurred in 60.3% of patients in the immediate VA-ECMO group and 61% in the early conservative group (risk difference −0.7, 95% CI −18.4 to 17.0). A numerically higher number of strokes (*n* = 3 vs. *n* = 0; *P* = 0.552), bleeding events (25 vs. 5; *P* = 0.067), and leg ischaemia (8 vs. 3; *P* = 0.999) was observed in the immediate VA-ECMO therapy group (*Table 2*). However, the study was not adequately powered to detect a statistical significance due to the small sample size. The incidence of serious adverse events was similar in those treated with VA-ECMO in any of the arms and those in the early conservative group who did not receive VA-ECMO.

#### Commentary

The ECMO-CS failed to show that immediate use of VA-ECMO in patients with severe or rapidly deteriorating CS improved outcomes compared with an early conservative approach. However, serious trial limitations may have affected the outcome. First, the cross-over of 23 patients (39%) from early conservative therapy to VA-ECMO implantation makes interpretation of the results challenging. Hence, the major limitation of this trial is that it actually evaluated the potential benefit of an early VA-ECMO strategy compared with VA-ECMO with initial conservative strategy. Moreover, the indication for downstream use of VA-ECMO in the early conservative therapy group was defined uniquely by a rise in lactate level—which cannot alone accurately portray haemodynamic deterioration. In fact, lactate levels are influenced by exogenous epinephrine administration (5.1% of patients in the early conservative group) and blood clearance methods, such as renal replacement therapy (5.2% of patients).^10^ Second, the trial was powered to detect a difference in the composite primary outcome and was, therefore, limited in detecting a statistical significance in individual outcomes of the primary endpoints, secondary endpoints, and subgroup analyses. Moreover, the sample size was calculated to detect a 50% reduction of the primary endpoint, a large predicted effect size. Larger studies are required to evaluate clinically relevant but somewhat smaller degree of risk reduction with VA-ECMO in patients with CS. Third, the similar incidence of adverse events between both groups, notably vascular and bleeding complications, could have been influenced by the high cross-over rate and the limited sample size, underestimating device-related complications. Finally, offloading strategy of the left ventricle (LV) through other MCS systems, such as intra-aortic balloon pump (IABP) or Impella, was not standardized and could have affected treatment results given their potential benefit in promoting myocardial recovery.^11^

### EURO-SHOCK

The EURO-SHOCK trial by Banning *et al*.^8^ was a multicentre, pan-European RCT conducted in 6 countries across 15 different centres that aimed to determine whether VA-ECMO could improve outcomes in patients with persistent CS 30 min after primary PCI of the culprit lesion. Persistent CS was defined as the presence of SBP <90 mmHg or maintained >90 mmHg with the addition of vasopressor or inotropic support, with evidence of hypoperfusion (*Table 1*). The study aimed to recruit 428 patients, but the overall enrolment rate was significantly impacted by the COVID-19 pandemic. A total of 35 patients were randomized to the VA-ECMO (*n* = 17) or to the standard therapy (*n* = 18) group. Intra-aortic balloon pump use was permitted as a means of LV unloading in those receiving VA-ECMO and as an MCS device in the standard therapy group. In the control group, the use of other MCS devices was accepted but acknowledged as protocol violation. A total of 428 patients were set to be included to detect a 27.5% reduction in the primary endpoint measure of all-cause mortality at 30 days. This was based on an anticipated 30-day mortality of 50% in the standard therapy group. The primary analysis was preformed according to the intention-to-treat principle. A pre-specified secondary as-treated analysis was also performed according to whether the patients received early VA-ECMO or not.

Baseline characteristics were similar between both groups. The median age was 67 years in the standard therapy group and 68 years in the VA-ECMO group. A total of 85% of patients were males. The extent of LV dysfunction and LVEF were similar between both groups. The primary endpoint measure of 30-day all-cause mortality occurred in 43.8% of patients in the VA-ECMO group (*n* = 7) and 61.1% (*n* = 11) of patients in the standard therapy group (HR 0.56, 95% CI 0.21–1.35; *P* = 0.22). Secondary endpoints of all-cause death (*n* = 7 vs. *n* = 13), cardiovascular death (*n* = 2 vs. *n* = 6), ischaemic stroke (*n* = 0 vs. *n* = 2), and recurrent myocardial infarction (*n* = 0 vs. *n* = 2) occurred numerically less frequently in the VA-ECMO group without any statistical significance. However, numerically higher rates of vascular complications (*n* = 3 vs. *n* = 0) and bleeding events (*n* = 5 vs. *n* = 1) occurred in the VA-ECMO group (*Table 2*). One-year all-cause mortality was 51.8% in the VA-ECMO group and 81.5% in the standard therapy arm (HR 0.52, 95% CI 0.21–1.26; *P* = 0.14). All-cause mortality at 12 months was numerically lower in the VA-ECMO group (*n* = 8, 59.8%) compared with the standard therapy group (*n* = 14, 81.5%; HR 0.52, 95% CI 0.21–1.26; *P* = 0.14).

#### Commentary

The EURO-SHOCK trial showed that in patients with persistent CS 30 min after primary PCI, VA-ECMO implantation resulted in numerically lower rates of 30 days and 1 year mortality. However, the study was significantly underpowered for the primary outcome as <10% of initially planned recruitment was completed. Therefore, no definite conclusions could be drawn from the data presented. Moreover, a significant cross-over rate between groups occurred which may have further affected treatment results; 5 out of the 17 patients randomized to the VA-ECMO group were never treated due to vascular complications or patient refusal. Interestingly, this study demonstrated a higher complication rate of vascular and bleeding events in the VA-ECMO group. Despite the challenges in interpreting these results, the study further emphasized the necessity in designing adequately powered trial to assess efficacy of VA-ECMO in this patient population and evaluate the potential risks associated with its implantation.

### ECLS-SHOCK

The ECLS-SHOCK trial by Thiele *et al*. was a pivotal large multicentre open-label RCT conducted in Germany and Slovenia across 15 different centres. It aimed to determine whether patients with AMI-CS defined as SCAI Stage C, D, or E with planned revascularization would benefit from early unselective VA-ECMO implantation in addition to usual medical therapy compared with the usual medical therapy alone.^9^ Cardiogenic shock was defined as SBP <90 mmHg for >30 min or the initiation of catecholamines to maintain SBP >90 mmHg, an arterial lactate of >3 mmol/L, and signs of impaired organ perfusion (*Table 1*). Patients, who had undergone CPR for over 45 min before randomization, had a mechanical cause of CS or had severe peripheral artery disease precluding insertion of VA-ECMO cannula were excluded from the trial. A total of 417 patients were randomized to VA-ECMO plus usual medical therapy and revascularization (*n* = 209) or usual medical therapy and revascularization (*n* = 208) with escalation to another MCS (IABP, Impella, or tandem heart) if deemed necessary. Patients in the control group were allowed to cross-over to the VA-ECMO group in case of specific predefined criteria for haemodynamic deterioration despite escalation of medical therapy and use of other MCS. These criteria included severe haemodynamic instability with impending haemodynamic collapse, an increase in arterial lactate of >3 mmol/L during a 6 h period, or an increase in vasopressor use by 50% from baseline to maintain a mean arterial pressure (MAP) >65 mmHg. The trial was designed to detect a 14% difference in the primary endpoint of death from any cause at 30 days. The primary analysis was performed according to the intention-to-treat principle. Relative risk for the primary outcome was performed for predefined subgroup analyses, and post hoc subgroup analysis of resuscitation vs. no resuscitation before randomization was performed.

Baseline characteristics were similar in both groups. The median age was 62 years in the VA-ECMO group and 63 years in the control group. A total of 81% of patients included were males. Over 66% of patients presented with ST-elevation myocardial infarction, approximately two-third had multivessel disease and 77.7% of patients underwent CPR prior to randomization. The median time until the return of spontaneous circulation was identical in both groups (20 min). Revascularization was performed by PCI in 96.6% of patients. Immediate PCI of non-culprit lesions was performed in over 20% of patients in both groups. Treatment with VA-ECMO was initiated during the index angiography in 91.9% of cases in the VA-ECMO group. Venoarterial extracorporeal membrane oxygenation was not initiated in 8.1% of patients in the VA-ECMO group and implementation of at least one LV unloading strategy was reported in 5.8% of patients. In the control group, a total of 15.4% of patients (*n* = 28) received MCS other than VA-ECMO of whom 2 did not fulfil the predefined escalation criteria. An additional 12.5% of patients crossed over to the VA-ECMO therapy group.

The primary endpoint of death from any cause at 30 days occurred in 47.8% of patients in the VA-ECMO group and 49% of patients in the control group [relative risk (RR) 0.98, 95% CI 0.8–1.19; *P* = 0.81]. No difference in the primary endpoint of death was observed across all pre-specified subgroups. No difference in the secondary endpoints of duration of ICU stay and hospital lengths of stay were observed between both groups. Safety outcomes of both moderate or severe bleeding (23.4 vs. 9.6%, RR 2.44, 95% CI 1.5–3.95) and peripheral vascular complications requiring intervention (11.0 vs. 3.8%, RR 2.86, 95% CI 1.31–6.25) were significantly higher in the VA-ECMO group (*Table 2*). In the overall trial population, refractory CS was the main cause of death (53%) followed by brain injury (26%).

#### Commentary

Despite being powered to detect a 14% absolute reduction in mortality in the VA-ECMO group, the ECLS-SHOCK trial failed to demonstrate a benefit with early implantation of VA-ECMO therapy in patients with AMI-CS compared with usual medical therapy and revascularization. This pivotal trial provides outcomes that dispute current clinical practice and shed light on the risks related to device implantation. Despite being a well-conducted RCT, there are some important trial limitations to consider. First, a quarter of patients were older than 69 years in the VA-ECMO group which does not reflect current practice across the world, as age above 65 or 70 years is an exclusion criterion for implantation of VA-ECMO in many centres. Moreover, with 77% of patients undergoing CPR prior to randomization for a median time of 20 min, underlying poor neurologic status may also have halted the potential benefit of VA-ECMO in this select patient population. Finally, immediate PCI of non-culprit lesions was performed in over 20% of patients in each group, despite clear evidence demonstrating that in CS, PCI of the culprit lesion only compared with multivessel PCI is associated with a mortality benefit.^12^ Second, the high cross-over rate of patients to VA-ECMO group may underestimate the benefit of this intervention. Third, offloading strategies were only used in 5.8% of patients with VA-ECMO. Consequently, further trials are needed to evaluate efficacy of VA-ECMO with LV offloading strategies. While this trial provides robust evidence against the use of early VA-ECMO in patients with AMI-CS, it cannot eliminate the potential benefit of VA-ECMO in patients with refractory CS.

## Discussion

Despite differences in patient selection, nuances in trial conduction, and variability in trial endpoints, our review revealed that the four available trials have failed to demonstrate a mortality benefit with the use of VA-ECMO in AMI-CS. The absence of a mortality benefit across all trials is due largely to complex patient characteristics, lack of venting strategies, and high rate of device-related complications. What is more, the lack of clinical equipoise from clinicians, as evidenced by high cross-over rates to VA-ECMO, raises a concern while enrolling these patients.

The recent patient-level meta-analysis of all four trials showed that the lack of mortality benefit with the use of VA-ECMO in AMI-CS was consistent across all pre-specified subgroups of age, sex, cardiac arrest prior to randomization, and type or location of myocardial infarction (*Table 3*). An increased rate of bleeding [odds ratio (OR) 2.44, 95% CI 1.55–3.84] and peripheral ischaemic vascular complications (OR 3.53, 95% CI 1.7–7.4) were also noted.^5^

**Table 3:** Clinical outcomes at 30 days of the patient-level meta-analysis of all four discussed trials

	VA-ECMO (*n* = 284)	Standard therapy (*n* = 283)	Effect size (95% CI)
Primary outcome			
** **30-day all-cause mortality	129/282 (46%)	135/283 (48%)	OR 0.93 (0.66–1.29)
Secondary outcomes			
** **Moderate or severe bleeding (BARC type 3–5) within 30 days	70/282 (25%)	34/283 (12%)	OR 2.44 (1.55–3.84)
** **Stroke within 30 days	11/282 (4%)	8/283 (3%)	OR 1.41 (0.56–3.57)
** **Peripheral ischaemic vascular complication within 30 days	32/281 (11%)	10/283 (4%)	OR 3.53 (1.70–7.34)
Additional outcome			
** **Poor neurological outcome (CPC 3 or 4) in survivors	38/134 (28%)	33/133 (25%)	OR 1.20 (0.70–2.07)

Data are shown as *n*/*N* (%) where the denominator is the number of patients with valid data in the meta-analysis.

BARC, Bleeding Academic Research Consortium; CPC, Cerebral Performance Category; VA-ECMO, venoarterial extracorporeal membrane oxygenation.

Adapted from Zeymer *et al*.^13^

### Patient selection

Age and underlying neurologic status are two baseline characteristics that may have neutralized the benefit of VA-ECMO implantation in the trials discussed. While no specified age cut-off exists for VA-ECMO implantation per the extracorporeal life support organisation (ELSO) guidelines, a recent meta-analysis of 12 000 patients demonstrated that age above 60 years was associated with increasing mortality rate.^14^ The median age of patients included in the above trials ranging from 62 to 70 years may have interfered with the primary outcome. However, the majority of patients suffering from AMI-CS are above the age of 60 and have inherently more comorbidities—including diabetes mellitus, chronic kidney disease, and peripheral vascular disease—which predisposes them to increased risk of death and vascular complications.^15^

Moreover, the potential for cardiac arrest to cause varying degrees of anoxic brain injury should prompt separate analysis of outcomes in patients with concomitant cardiac arrest and CS.^16^ The utilization of MCS systems will not improve mortality in this patient population given competing neurologic injury. However, a significant proportion of patients included in the aforementioned trials had undergone resuscitation prior to randomization. While subgroup analyses did not demonstrate a difference in all-cause mortality, the studies were underpowered for that outcome. The inclusion of patients with unclear extent of neurologic injury may dilute the potential benefit seen from VA-ECMO implantation given their inherent worse prognosis.

In addition to baseline characteristics, adequate patient selection based on clinical and haemodynamic parameters is crucial to identify those with the highest chance of recovery or those who are candidates for destination therapy. In fact, placing a patient on VA-ECMO who would otherwise respond to conservative therapy may result in worse outcomes from device-related complications. Conversely, placing a patient on VA-ECMO who is in extremis and unlikely to achieve sufficient myocardial recovery is futile. The stages of shock according to the SCAI shock classification are helpful to select the optimal window for VA-ECMO implantation, with data suggesting that Stages C and D are the most appropriate.^17,18^ Frequent reassessment of patients in SCAI Stage B is critical to ensure timely insertion of VA-ECMO and prevent worsening of the metabolic cascade. In fact, pre-cannulation SCAI shock classification has been demonstrated to be a strong predictor of in-hospital mortality and can help physicians prognosticate and guide further interventions.^17^ Adequate patient selection remains complex, requiring careful assessment by the HEART team of the clinical and haemodynamic parameters as well as establishing long-term goals.

### Timing of venoarterial extracorporeal membrane oxygenation implantation in cardiogenic shock

The optimal timing of VA-ECMO implantation remains unclear and the window for the maximal benefit of this therapy appears narrow.^19^ Prompt revascularization can lead to rapid improvement in the LVEF in some patients, precluding the need for VA-ECMO. However, a small retrospective study of 253 patients noted a potential benefit of VA-ECMO implantation prior to PCI.^20^ These previously described RCTs all demonstrated no difference in mortality rates between the immediate and downstream use of VA-ECMO in patients with AMI-CS. In the EURO-SHOCK trial, downstream use of VA-ECMO only if there was persistent CS 30 minutes after primary PCI resulted in numerically lower rates of mortality. In the ECLS-SHOCK trial, no differences in outcomes were observed among those who underwent immediate VA-ECMO implantation prior (22%), during (26%), or after (52%) revascularization and those who underwent VA-ECMO implantation only in case of clinical deterioration.

Evidence suggests that early unselected VA-ECMO should not be performed in patients in AMI-CS because of the following: (i) the lack of benefits demonstrated in trials, (ii) the complications related to device implantation, and (iii) the potential improvement of CS after revascularization. It is reasonable to defer VA-ECMO implantation to only select cases of persistent CS despite medical and revascularization strategies and before irreversible end-organ damage occurs.

### Left ventricle unloading strategies

Peripheral VA-ECMO implantation results in increased LV afterload, which may hinder potential LV recovery due to increased LV wall stress and myocardial oxygen demand.^21^ To overcome these detrimental effects, several unloading strategies have been proposed, including IABP, Impella, and atrial septostomy.^21^ Although LV unloading devices might stabilize patients with CS, vascular and bleeding complications may mitigate their potential benefits. A recent multicentre cohort study demonstrated a 30-day mortality benefit with the use of Impella in those treated with VA-ECMO in AMI-CS at the expense of an increased risk of severe bleeding complications.^22^ A large-scale nationwide study demonstrated that the use of IABP in addition to VA-ECMO in patients with AMI-CS was associated with significantly lower 30-day mortality, without an increase in cerebral and major bleeding events.^11^ Consequently, the lack of benefits with VA-ECMO noted in these trials may be due to the infrequent adoption of unloading strategies.^23,24^ This hypothesis is being tested in multiple ongoing RCTs. The ANCHOR trial aims to compare VA-ECMO therapy with IABP to the standard of care in patients with AMI-CS (NCT04184635). The UNLOAD ECMO trial is set to determine whether VA-ECMO and Impella are superior to VA-ECMO alone in patients with CS (NCT05577195). Lastly, the REVERSE trial intents to evaluate whether VA-ECMO and Impella as a LV unloading strategy is superior to VA-ECMO alone in patients with CS (NCT03431467).

### Device-related complications

Bleeding and peripheral vascular complications remain a major limitation of VA-ECMO implantation hindering any potential cardiovascular benefits. Bleeding is associated with an increased risk of mortality in patients with AMI-CS.^25,26^ Apart from the use of larger access sheaths, the increased risk of bleeding is also due to increased consumption of coagulation factors and platelets from enhanced generalized inflammatory states.^25^ As patient-related risk factors are often non-modifiable, careful patient selection, safe femoral access techniques incorporating ultrasound guidance, and dedicated haemostasis protocols should be implemented in an attempt to decrease vascular complications and bleeding.

### Application to clinical practice

The presented literature of VA-ECMO in CS does not support its routine use in clinical practice. Although VA-ECMO provides maximal haemodynamic support, the lack of survival benefits and the increased vascular and bleeding complications highlight the importance of adequate patient selection. We have yet to identify which subset of patients would benefit from this intervention, but an approach incorporating patient comorbidities, likelihood of recovery, and haemodynamic parameters is necessary. Venoarterial extracorporeal membrane oxygenation should be considered in patients with AMI-CS who are deteriorating despite adequate medical therapy and revascularization. Early involvement of the HEART team is crucial. Assessment of comorbidities, neurologic statuses, and major contraindications, such as severe peripheral vascular disease, are the foremost steps. Evaluation of haemodynamic parameters, including lactate levels, cardiac index, and other perfusion parameters, is the subsequent step. If the patient is deemed to be a deteriorating SCAI shock Stage C or D, VA-ECMO implantation is reasonable. The use of LV unloading device should be encouraged to decrease the LV afterload. Importantly, VA-ECMO cannulation should ideally be done under ultrasound guidance to minimize vascular complications. If the patient has significant calcifications, it is reasonable to insert a distal perfusion catheter to avoid a peripheral ischaemic event. Daily assessment of the necessity of VA-ECMO is required.

## Conclusions

Despite their limitations, these trials suggest that an early VA-ECMO implantation strategy in patients with AMI-CS is not associated with a clinical benefit compared with standard therapy with VA-ECMO escalation in case of haemodynamic compromise. Vascular and bleeding complications from device implantation remain a major safety concern, particularly in patients with age-related comorbidities. The current role of VA-ECMO in AMI-CS remains unclear. Better patient selection, adoption of LV unloading strategies, and restriction of cross-over to the intervention group are key to designing adequately powered trials to properly assess the role of VA-ECMO in AMI-CS and guide clinical practice.

## Data Availability

No data were generated or analysed for or in support of this paper.

## Lead author biography

Dr Tara Gédéon is a cardiology fellow at McGill University, Canada. She received her Doctorate of Medicine from McGill University, where she also completed her Internal Medicine Training. Her current research interests include cardiovascular interventions, mechanical circulatory support, critical care, and women’s cardiovascular health.
